# Comprehensive molecular and clinical characterization of Asian melanoma patients treated with anti-PD-1 antibody

**DOI:** 10.1186/s12885-019-6030-5

**Published:** 2019-08-14

**Authors:** Jiyun Lee, Su Jin Lee, Kyung Kim, Seung Tae Kim, Kee-Taek Jang, Jeeyun Lee

**Affiliations:** 1 Division of Hematology-Oncology, Department of Medicine, Samsung Medical Center, Sungkyunkwan University School of Medicine, Seoul, South Korea; 2 Division of Hematology-Oncology, Department of Internal Medicine, Ewha Womans University College of Medicine, 260, Gonghang-daero, Gangseo-gu, 07804 Republic of Korea; 3 Department of Pathology and Translational Genomics, Samsung Medical Center, Sungkyunkwan University School of Medicine, Seoul, South Korea

**Keywords:** Melanoma, PD-1, Genomic, Biomarker

## Abstract

**Background:**

Clinical features of Asian melanoma patients are distinct from those of Western patients. This study was designed to determine the molecular and clinical characteristics of Asian melanoma patients treated with anti-PD-1 antibody.

**Methods:**

Patients with recurrent or metastatic melanoma who began anti-PD-1 antibody therapy between January 2015 and April 2018 were retrospectively reviewed. Patients who underwent next-generation sequencing were also analyzed.

**Results:**

A total of 152 patients were included. The median age was 61 years, and 53% of patients were female. A total of 56 patients (37%) received immunotherapy as second-line or greater chemotherapy. Primary sites were acral (38%), mucosal (31%), cutaneous (24%), uveal (2%), and unknown (5%). The overall response rate was 17% (95% CI, 11–22%), and disease control rate was 60% (95% CI, 52–68%). The median progression-free survival (PFS) was 4.2 months (95% CI, 1.8–6.6 months), and median overall survival (OS) was 32.9 months (95% CI, 20.0–45.7 months). However, *BRAF*^*V600*^ and *KIT* mutational statuses were not associated with response or survival. High neutrophil-lymphocyte ratio (NLR) was associated with poor PFS (median PFS 6.9 vs. 2.4 months, *p* = 0.015) and OS (median OS NR vs. 10.4 months, *p* < 0.001). In multivariate analysis, high NLR independently predicted poor survival.

**Conclusion:**

This study includes the largest set of integrated genomic data analyzing Asian patients with melanoma treated with immunotherapy. *BRAF* ^*V600*^ and *KIT* mutational statuses were not associated with response or survival, and high NLR was a strong predictor of poor response to and survival with anti-PD-1 therapy.

## Background

The role of anti-PD-1 therapy is well established in malignant melanoma – pembrolizumab and nivolumab have been approved as first-line therapy in advanced melanoma. Such advances in immunotherapy have significantly improved the response rates and survival outcomes in patients with advanced melanoma [1, 2]. Melanoma was traditionally classified based on histologic growth pattern – superficial spreading, lentigo maligna, nodular, and acral lentiginous melanoma. However, with increasing data on distinct molecular aberrations and primary locations, a novel classification has been proposed – cutaneous (with or without chronic sun-induced damage), acral, and mucosal melanoma. Previous studies have shown that acral and mucosal melanoma, which have higher frequency of *KIT* mutation [3, 4], are the most prevalent subtypes in Asian populations [5, 6]. Conversely, cutaneous melanoma is the predominant subtype in Caucasian populations, which have higher incidence of *BRAF* mutation [4, 7].

Despite the increasing incidence of malignant melanoma in Asia, the absolute incidence remains small [6, 8, 9], and there are limited data available on immunotherapy treatment outcomes in Asian patients with melanoma. The effect of mutation status on response to immunotherapy is poorly understood. Despite the role of anti-PD-1 therapy as a first-line agent, the use of biomarkers for patient selection is an area of ongoing debate. In search of a readily available biomarker, the ratio of neutrophils to lymphocytes (NLR) has been evaluated in many solid cancers, including melanoma [10–12], and has emerged as an important biomarker to predict response to immunotherapy.

The aim of the present study was to evaluate the treatment efficacy of anti-PD-1 therapy in Asian patients with melanoma. Additionally, we sought biomarkers to predict treatment response to anti-PD-1 antibody in patients with melanoma.

## Methods

### Patients

A total of 152 consecutive patients with recurrent or metastatic melanoma who began anti-PD-1 (nivolumab or pembrolizumab) therapy between January 2015 and April 2018 were retrospectively analyzed. Baseline characteristics including age, sex, ECOG performance status, previous therapies, melanoma subtype, disease stage, metastatic sites, baseline CBC, LDH, treatment response, adverse events, and survival outcomes were obtained through medical records and tumor imaging review. This study was approved by the Institutional Review Board of Samsung Medical Center (IRB No. 2018–07-080), and informed consent was waived. All genomic analyses using cancer panel were used with consent.

### Treatment and response

All patients received pembrolizumab 2 mg/kg IV every 3 weeks or nivolumab 3 mg/kg IV every 2 weeks until progression, unacceptable toxicity, or patient refusal. Patients were evaluated at baseline and every 6–9 weeks after starting treatment. Response categories were assessed using RECIST 1.1 [13]. In addition to response defined by RECIST, efficacy was also defined by durable clinical benefit (DCB), which included complete response (CR), partial response (PR), and stable disease (SD) lasting for more than 6 months. Adverse events were graded based on the National Cancer Institute Common Terminology Criteria for Adverse Events version 5.0 (CTCAE v5.0, National Cancer Institute, Bethesda, MD, USA).

### Next-generation sequencing (NGS)

Next-generation sequencing (NGS) was performed on formalin-fixed, paraffin-embedded specimens using an extensively validated platform (Oncomine™ Comprehensive Assay v1, ThermoFisher Scientific, Waltham, MA, USA; www.thermofisher.com). Methods for DNA extraction and sequencing have been extensively validated and published [14].

### Statistical analysis

Descriptive statistics were used to summarize patient and treatment characteristics. NLR was defined as the quotient of baseline absolute neutrophil count divided by absolute lymphocyte count. Each nominal variable was compared using Fisher’s exact test or *X*^*2*^-test. PFS was defined as the time from initiation of anti-PD-1 therapy to documentation of disease progression or death. OS was defined as the time from initiation of anti-PD-1 therapy to death from any cause. Survival curves of categorical variables were calculated using the Kaplan-Meier method and compared using the log-rank test.

Univariate/multivariate models of patients for tumor characteristics in association with PFS and OS were based on Cox proportional hazards regression analyses. Results were presented as hazard ratio (HR) with 95% confidence interval (CI). A *p*-value less than 0.05 was considered statistically significant, and all analyses were performed using IBM SPSS Statistics 24 (Armonk, NY, USA).

## Results

### Patient characteristics

A total of 152 consecutive patients was treated with anti-PD-1 therapy. Baseline patient characteristics are summarized in Table 1. The median age was 61 years (range 21–82), and 80 patients (53%) were female. There were 58 patients (38%) with acral subtype, 47 (31%) with mucosal subtype, 36 (24%) with cutaneous subtype, 4 (2%) with uveal subtype, and 7 (5%) with unknown primary. M staging was based on cutaneous melanoma criteria for all patients– 42 (28%) with stage M1c and 11 (7%) with stage M1d (with brain metastases). A total of 32 patients (21%) had elevated baseline LDH, and 78 patients (52%) had elevated baseline NLR (≥2.10). Nivolumab was received by 30% of patients, and pembrolizumab was received by 70% of patients. A total of 56 (37%) patients were previously treated with at least one systemic therapy, including cytotoxic chemotherapy, ipilimumab, interleukin-2, or *BRAF*/*MEK* inhibitors.

**Table 1:** Baseline Characteristics

	No. (%)
Total N	152 (100)
Median age (range), years	61 (21–82)
Sex	
Male	72 (47%)
Female	80 (53%)
Performance status	
ECOG 0–1	149 (98%)
ECOG ≥2	3 (2%)
Subtypes	
Acral	58 (38%)
Mucosal	47 (31%)
Cutaneous	36 (24%)
Uveal	4 (2%)
Other (unclassifiable)	7 (5%)
M staging of extent of metastasis	
M0	23 (15%)
M1a	38 (25%)
M1b	38 (25%)
M1c	42 (28%)
M1d	11 (7%)
BRAF^V600^ status (*n* = 133)	
Mutant	23/133 (17%)
Wildtype	110/133 (83%)
KIT status (*n* = 98)	
Mutant	14/98 (14%)
Wildtype	84/98 (86%)
Lactate dehydrogenase concentration	
Normal	101 (66%)
Elevated	32 (21%)
Unknown	19 (13%)
Number of lines of previous systemic therapies	
0	96 (63%)
1	28 (18%)
2	22 (15%)
≥3	6 (4%)
Type of previous treatment	
Ipilimumab	17 (11%)
Interleukin-2	2 (1%)
BRAF/MEK inhibitor	6 (4%)
Cytotoxic chemotherapy	45 (30%)

*BRAF*^*V600*^ and *KIT* mutational statuses were evaluated in 133 and 98 patients, respectively, including 59 patients who underwent NGS. The incidence of *BRAF*^*V600*^ and *KIT* mutants was 23 of 133 patients (17%) and 14 of 98 patients (14%), respectively.

Data was last collected on 25 June 2018. The median follow-up duration was 18.8 months (range 3.0–42.3 months), and 25 (16%) patients were still receiving anti-PD-1 therapy. The most common reason for treatment discontinuation was disease progression in 92 (61%) patients, followed by disease stabilization/regression (*n* = 22, 14%), loss to further visits (*n* = 12, 8%), and adverse events (n = 1). The median treatment duration was 2.6 months (range 0.5–32.5 months).

### Response and survival

The overall response rate (ORR) and disease control rate (DCR) were 17% (95% CI, 11–22%) and 60% (95% CI, 52–68%), respectively (Table 2). The median time to response was 2.0 months, and the median duration of response was 6.2 months. The median OS was 32.9 months (95% CI, 20.0–45.7 months), and the median PFS was 4.2 months (95% CI, 1.8–6.6 months). There was no significant difference in response rates or survival outcomes according to type of anti-PD-1 therapy received.

**Table 2:** Responses to immunotherapy

RECIST v1.1	All treated patients (*n* = 152)
Best response	
Complete response	4 (3%)
Partial response	22 (14%)
Stable disease	66 (43%)
Progressive disease	54 (36%)
Not evaluable	6 (4%)
Overall response rate	17% (95% CI, 11–22)
Disease control rate	60% (95% CI, 52–68)
Median time to response	2.0 months
Median duration of response	6.2 months

Patients with *BRAF*^*V600*^ mutant (m*BRAF*^*V600*^) who were previously treated with *BRAF*/*MEK* inhibitors demonstrated poor PFS (1.2 vs. 8.0 months, *p* = 0.039) and OS (1.2 vs. 32.9 months, *p* = 0.002) compared to patients with m*BRAF*^*V600*^ and no previous therapy with *BRAF*/*MEK* inhibitors. No patients with m*BRAF*^*V600*^ who underwent previous *BRAF/MEK* inhibitor treatment demonstrated a clinical response to anti-PD-1 therapy.

### Efficacy analysis according to neutrophil-to-lymphocyte ratio (NLR)

**Fig. 1:**
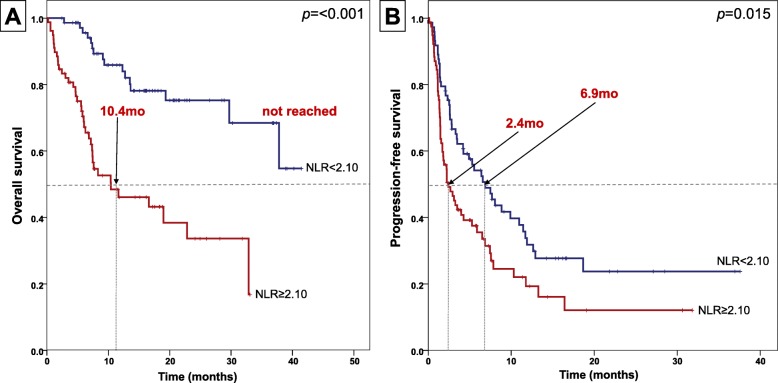
Kaplan-Meier survival curves for (**a**) overall survival and (**b**) progression-free survival according to baseline neutrophil-lymphocyte ratio

The median NLR was 2.1 (0.6–188.8). High NLR was defined as a value greater than or equal to the median value (2.1). Low NLR (< 2.10), which was observed in 73 patients (48%), showed superior PFS (median 6.9 vs. 2.4 months, *p* = 0.015) and OS (median not reached vs. 10.4 months, *p* < 0.001) (Fig. 1). Patients with low NLR also demonstrated a superior DCR (59.8 vs. 30.5%, *p* < 0.001) and DCB (59.0 vs. 41.1%, *p* = 0.033).

### Genomic analysis

**Fig. 2:**
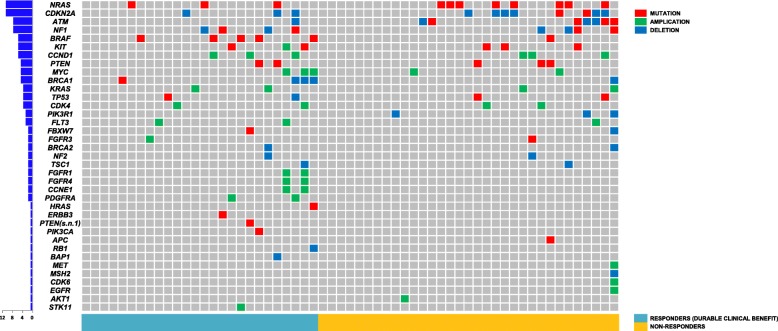
Genomic landscape of 59 patients with melanoma. *NRAS* mutation and *CDKN2A* deletion were the most commonly detected mutations, with no statistically significant difference between responder and non-responder groups. Neither *BRAF*^V600^ nor *KIT* mutational status affected treatment outcome of immunotherapy

Genomic landscapes of 59 patients are shown in Fig. 2. Among the most commonly detected mutations were *NRAS* mutation (11/59, 19%), *CDKN2A* deletion (9/59, 15%), *CCND1* amplification (6/59, 10%), *MYC* amplification (5/59, 8%), and *CDK4* amplification (4/59, 7%). Response rate was not associated with *BRAF*^*V600*^ (ORR 19 vs. 13%, *p* = 0.493; DCR 65 vs. 44%, *p* = 0.060; DCB 42 vs. 39%, *p* = 0.812) or *KIT* (ORR 16 vs. 29%, *p* = 0.231; DCR 64 vs. 57%, *p* = 0.608; DCB 42 vs. 43%, *p* = 0.933) mutational status.

*NRAS* mutational status did not have a statistically significant effect on response rate (ORR 20.8 vs. 9.1%, *p* = 0.670; DCR 77.1 vs. 72.7%, *p* = 0.712) or survival outcome (PFS 7.7 vs. 5.5 months, *p* = 0.361; OS not reached in either arm). Similarly, the response rates (ORR 20.0 vs. 11.1%, *p* = 1.000; DCR 76.0 vs. 77.8%, *p* = 1.000) and survival outcomes (PFS 7.7 vs. 5.2 months, *p* = 0.489; OS not reached in either arm) did not differ according to *CDKN2A* deletion status.

### Cox regression models on survival outcomes

Univariate and multivariate analyses using a Cox model were performed including NLR in addition to potential prognostic factors of age, ECOG PS, histologic subtype, M stage, mutational status, and LDH (Table 3). Low LDH level and low NLR (< 2.10) level were associated with superior PFS and OS on both univariate and multivariate analyses. *BRAF*^V600^ mutant was associated with poorer OS on univariate analysis (HR 2.232, *p* = 0.018) but not on multivariate analysis (HR 2.014, *p* = 0.101).

**Table 3:** Univariate and multivariate analyses of survival by baseline characteristics

	PFS				OS			
	Univariate analysis		Multivariate analysis		Univariate analysis		Multivariate analysis	
	HR	*p*-value	HR	*p*-value	HR	*p*-value	HR	*p*-value
Age								
<60	1	0.095			10.686	0.162		
≥60	0.722 (0.492–1.059)				(0.404–1.164)			
Performance status								
ECOG < 2	1	0.059	1	0.819	1	**0.002**	1	0.565
ECOG ≥2	3.067 (0.957–9.836)		1.160 (0.325–4.132)		6.750 (2.068–22.026)		1.494 (0.381–5.858)	
Subtypes								
Mucosal	1	0.375			1	0.503	–	
Cutaneous	1.182 (0.687–2.033)	0.400			0.954 (0.503–1.810)	0.885		
Acral	1.221 (0.767–1.944)	0.545			1.130 (0.548–2.329)	0.741		
Uveal	2.506 (0.877–7.157)	0.086			2.407 (0.703–8.243)	0.162		
Subtypes								
Non-cutaneous	11.233	0.329	–	–	11.045	0.879	–	–
Cutaneous	(0.810–1.879)				(0.591–1.848)			
M stage								
M1a	1	**<0.001**	1	**0.001**	1	**<0.001**	1	**0.017**
M1b	1.751 (1.002–3.060)	**0.049**	2.032 (1.133–3.642)	**0.017**	1.830 (0.756–4.430)	0.180	1.769 (0.590–5.307)	0.309
M1c	2.524 (1.466–4.344)	**0.001**	2.257 (1.279–3.984)	**0.005**	4.659 (2.109–10.294)	**<0.001**	3.945 (1.075–14.474)	**0.039**
M1d	4.495 (2.073–9.747)	**<0.001**	5.167 (2.24–11.900)	**< 0.001**	5.753 (2.142–15.451)	**0.001**	8.838 (2.192–35.626)	**0.002**
Bone metastasis								
No	1	**0.005**	1	0.280	1	**0.011**	1	0.108
Yes	1.959 (1.219–3.149)		1.370 (0.775–2.422)		2.154 (1.190–3.898)		1.995 (0.860–4.629)	
Liver metastasis								
No	1	**<0.001**	1	0.644	1	**<0.001**	1	0.695
Yes	2.228 (1.452–3.420)		0.835 (0.389–1.795)		3.687 (2.177–6.247)		0.795 (0.253–2.502)	
*BRAF*^*V600*^ status								
Wildtype	1	0.431	–	–	1	**0.018**	1	0.101
Mutant	1.242 (0.724–2.133)				2.232 (1.150–4.330)		2.014 (0.872–4.655)	
*KIT* status								
Wildtype	1	0.888	–	–	1	0.333	–	–
Mutant	1.052 (0.519–2.131)				1.557 (0.635–3.818)			
LDH								
Normal	1	**0.001**	1	**0.035**	1	**<0.001**	1	**0.011**
Elevated	2.149 (1.361–3.393)		1.723 (1.039–2.860)		4.476 (2.512–7.974)		2.676 (1.248–5.736)	
NLR								
< 2.10	1	**0.017**	1	**0.009**	1	**<0.001**	1	**<0.001**
≥ 2.10	1.606 (1.090–2.367)		1.802 (1.160–2.799)		4.103 (2.243–7.504)		4.583 (2.121–9.907)	

*PFS* progression-free survival, *OS* overall survival, *NRL* neutrophil/lymphocyte ratio, *LDH* lactate dehydrogenase concentration

The numbers in boldface represented the values with statistical significance

### Adverse events

The most common adverse event related to anti-PD-1 therapy was pruritus (19%, 29/152), followed by anorexia (15%, 22/152), skin rash (13%, 20/152), and fatigue (13%, 19/152). Two patients experienced serious adverse events of grade 3 or 4, which led to permanent treatment discontinuation in one case with myasthenia gravis (Table 4).

**Table 4:** Treatment-related adverse events

	Grade		
	1	2	3–4
Pruritus	20 (13%)	9 (6%)	–
Anorexia	19 (13%)	3 (2%)	–
Skin rash	13 (9%)	7 (5%)	–
Fatigue	13 (9%)	6 (4%)	–
Nausea	15 (10%)	2 (1%)	–
Cough	10 (6%)	1 (< 1%)	–
Insomnia	6 (4%)	1 (< 1%)	–
Hypothyroidism	–	5 (3%)	–
Hypopigmentation	4 (3%)	–	–
Diarrhea	4 (3%)	1 (< 1%)	–
Hypopituitarism	–	1 (< 1%)	–
Hyperglycemia	–	–	1 (<1%)
Myasthenia gravis	–	–	1 (<1%)^a^

^a^The only case with treatment discontinuation

## Discussion

To our knowledge, this is the largest integrated genomic analysis of Asian patients with melanoma treated with anti-PD-1 therapy. In this study, the ORR and DCR were 17% (95% CI, 11–22%) and 60% (95% CI, 52–68%), respectively. These values are slightly lower than those of previous prospective studies, which reported ORR ranging from 21 to 40%. However, our study included patients with poor performance status and brain metastases. This is more representative of patients with advanced melanoma in the clinical setting and possibly explains the low response rates. The median PFS and OS of 4.2 and 32.9 months, respectively, were comparable to those of Caucasian patients [1, 2, 15, 16]. These findings suggest that immune checkpoint blockades are viable treatment options for Asian patients, especially considering the limited utilization of *BRAF*/*MEK* inhibitors due to low *BRAF*^*V600*^ mutant prevalence.

In exploratory analysis, m*BRAF*^*V600*^ patients who were previously treated with *BRAF*/*MEK* inhibitors were analyzed for response rates and survival outcomes. They demonstrated poor PFS and OS compared to m*BRAF*^*V600*^ patients without previous therapy with *BRAF*/*MEK* inhibitors. It is also notable that no m*BRAF*^*V600*^ patients with previous *BRAF*/*MEK* inhibitor therapy experienced a clinical response to immunotherapy. Although we should be cautious about drawing conclusions from a small subset population, the results suggest that early immunotherapy in m*BRAF*^*V600*^ patients is more effective than early targeted therapies. Two randomized trials (NCT02224781 and NCT02631447) are currently investigating the optimal treatment sequence in a larger m*BRAF*^*V600*^ cohort, and the results are highly awaited.

The incidence of *KIT* mutations in our study population was relatively low compared to that of previous reports [3, 17]. This is due to the low accessibility of the *c-KIT* inhibitor imatinib in the real-world setting. Consequently, clinicians are reluctant to pursue genomic testing for *KIT* mutational status.

*NRAS* mutation was detected in 19% of our cohort who underwent NGS, an incidence similar to that of previous reports [18]. Johnson et al. reported that *NRAS*-mutant patients demonstrate a superior response to immunotherapy (ORR 32 vs. 20%, *p* = 0.07), particularly to anti-PD-1 and anti-PD-L1 agents (ORR 64 vs. 30%, *p*-value not provided), which is possibly related to higher PD-L1 expression [19]. Horn et al. reported that melanoma cell lines with chromosomal loss of *CDKN2A* associated with *JAK2* deletion are prone to immunotherapy resistance. However, neither *NRAS* mutation nor *CDKN2A* deletion status had statistically significant effects on response rate or survival outcome with anti-PD-1 therapy in our cohort. Whether this conflicting outcome is related to ethnic differences requires further validation in future prospective studies.

Studies regarding response outcomes to immunotherapy according to PD-L1-positivity are confounding [1, 15]. There is an increasing need for an easily accessible and affordable biomarker to predict anti-PD-1 therapy responses, and NLR has emerged as a promising option. The tumor microenvironment is characterized by chronic inflammation, and neutrophils reflect the host inflammatory status in patients with cancer [20]. Although their role is multifactorial, neutrophils have been shown to contribute to tumor initiation, angiogenesis, proliferation, and metastatic spread [21]. However, the utility of NLR as a predictive biomarker in Asian patients with melanoma has not been previously validated.

In our study, NLR was the only independent factor, other than LDH, associated with superior PFS and OS on both univariate and multivariate Cox regression analyses. This is consistent with findings from previous studies [22–24], highlighting the role of NLR as an important biomarker to predict response and survival outcomes to immunotherapy in patients with melanoma. A cut-off value of 2.10 was utilized in this study; however, similar trends were demonstrated with cut-off values of 2.0, 3.0, and 5.0. Further studies are needed to validate the best cut-off value for utilization of NLR.

## Conclusion

This study is the largest integrated genomic analysis of Asian patients with melanoma treated with anti-PD-1 therapy. The heterogeneous patient population in our study reflects the real-world efficacy and safety of anti-PD-1 therapy in patients with advanced melanoma. Despite different distributions in subtypes, *BRAF*^V600^ or *KIT* mutational status does not affect response to immunotherapy. Low NLR is a strong predictor of higher response and longer survival in response to immunotherapy, and it may be useful as a biomarker.

## Data Availability

The datasets used and/or analyzed during the current study are available from the corresponding author on reasonable request.

## Abbreviations

CR: complete response
DCB: durable clinical benefit
DCR: disease control rate
HR: hazard ratio
NGS: next-generation sequencing
NLR: neutrophil/lymphocyte ratio
ORR: overall response rate
OS: overall survival
PFS: progression-free survival
PR: partial response
SD: stable disease
